# Long-term wastewater surveillance of SARS-CoV-2: a 5.5-year study reveals correlation with public health indicators and early warning potential during and after the pandemic

**DOI:** 10.3389/fmicb.2026.1854041

**Published:** 2026-08-18

**Authors:** Mahé Liabeuf, Mylène Toubiana, Charlotte Arnal, Emmanuel Soyeux, Vincent Foulongne, Patricia Licznar-Fajardo, Olivier Courot, Patrick Monfort, Estelle Jumas-Bilak

**Affiliations:** 1 HSM, Univ Montpellier, CNRS, IRD, Montpellier, France; 2 Veolia Scientific & Technological Expertise Department, Aubervilliers, France; 3 PCCEI, Univ Montpellier, CNRS, IRD, Montpellier, France; 4 Virology Laboratory, CHU de Montpellier, Montpellier, France; 5 SPIR, CHU de Montpellier, Montpellier, France; 6 IAGE, Montpellier, France

**Keywords:** wastewater-based epidemiology, SARS-CoV-2, COVID-19, RT-dPCR, long-term19 surveillance, public-health indicators, pandemics, endemicity

## Abstract

**Background:**

While wastewater surveillance (WS) is a validated tool for SARS-CoV-2 monitoring, its long-term reliability and forecasting accuracy remain debated. This study, spanning a 65-month period (5.5 years), evaluates the dynamics of SARS-CoV-2 in municipal wastewater across multiple epidemiological shifts, including vaccination rollouts, variant emergence, and change in testing policies.

**Materials and methods:**

Inlet wastewater from a large metropolitan area in southern France (450,000 inhabitants) was monitored weekly or twice weekly. SARS-CoV-2 RNA (IP4 target) was quantified by Reverse Transcription digital PCR (RT-dPCR) in 345 samples and compared to 15 regional public health indicators (virological, hospital-based, and syndromic) using Spearman correlation coefficients (*r_s_*). The time lags in days that maximized *r_s_* were calculated using a lag-based association analysis to evaluate the early warning capacity of WS.

**Results:**

Overall, the SARS-CoV-2 RNA load in wastewater was consistently correlated with specific virological indicators such as incidence rate, and non-specific syndromic indicator such as emergency procedures. WS of SARS-CoV-2 RNA demonstrated significant early warning capabilities, preceding hospital admissions by 8 to 12 days and mortality by up to 30 days during the pandemic phase. However, the strength of the correlations and the early warning capacity varied across epidemiological phases, the outbreak wave, the variants circulating and the population’s vaccination rate. Crucially, in the post-pandemic period, WS remained a robust proxy and could provide arguments about the virus causing an increase in syndromic indicators.

**Conclusion:**

This 5.5-year longitudinal study provides one of the most comprehensive datasets of SARS-CoV-2 environmental dynamics. It advocates for the permanent implementation of WS to monitor respiratory and emerging infectious threats in a post-pandemic world.

## 1 Introduction

Since its emergence in late 2019, SARS-CoV-2 has caused nearly 700 million confirmed infections, resulting in approximately 1% of global deaths (World Health Organization, 2025). By 5 May 2023, World Health Organisation (WHO) ended the COVID-19 Public Health Emergency of International Concern, and further studies confirmed a decline in morbidity as the disease transitioned to an endemic state (Bartha et al., 2024). Current COVID-19 incidence trends are characterized by a series of peaks and troughs, with timing and patterns varying between countries and regions. Epidemic patterns and seasonality remain unclear, with unexpected epidemic peaks still occurring (Contreras et al., 2023). Understanding and predicting the dynamics of SARS-CoV-2 is crucial for implementing effective public health interventions, such as gradual rises and drops, to align policies with viral circulation. Non-pharmaceutical interventions and vaccination are indeed essential to mitigate the impact of the virus, but they must be promoted in a timely and appropriate manner through active surveillance (Contreras et al., 2023).

During pandemics, monitoring the wastewater concentration of SARS-CoV-2 RNA at the entrance of wastewater treatment plants (WWTP) or upstream, i.e., wastewater surveillance (WS), has proven effective for gathering information about SARS-CoV-2 shedding in the population, including asymptomatic subpopulations (Shah et al., 2022). Particularly, the concentration of SARS-CoV-2 RNA in wastewater correlate with the incidence of COVID-19 in France (Lazuka et al., 2021), Spain (López-Peñalver et al., 2023), China (Lv et al., 2025), Japan (Tanimoto et al., 2022), Africa (Dzinamarira et al., 2022) and the USA (Duvallet et al., 2022). These are few examples from the large body of literature about WS of COVID-19 across diverse countries and cities. Several studies suggested that one of the major advantages of WS is its predictive signal compared to the detection of individual cases of the virus in a population (Doss et al., 2025; López-Peñalver et al., 2023).

The correlation with clinical cases and the predictiveness of WS have been described as virus- and variant-dependent (Armas et al., 2023; Yaniv et al., 2021). However, these correlations may also be influenced by factors such as the epidemic phase (Li et al., 2023), population immunity, and vaccination coverage (Armas et al., 2023; Yaniv et al., 2021), as well as public health policies, such as the availability of screening tests. Few studies have focused on the post-pandemic period, during which SARS-CoV-2 is expected to circulate endemically. These studies have also suggested that WS could inform the public health response (Wei et al., 2026; Ulloa-Medina et al., 2026; Daniel et al., 2025; Wang et al., 2025). However, since most studies on SARS-CoV-2 WS have focused on the pandemic and/or epidemic periods, it remains unclear whether the correlations and predictive value persist under changing epidemiological and surveillance conditions, such as vaccination, variant emergence, reduced testing and endemic transmission. Long-term surveillance studies are needed, and few have examined both the pandemic and post-pandemic periods (Kagami et al., 2025).

For this study, we collected 345 samples of inlet wastewater from the WWTP in Montpellier, the main metropolitan area in the Hérault department in southern France, which has a population of around 450,000 — representing more than one third of the total population of the department. The study period covered 35 months before and 30 months after the official end of the Public Health Emergency of International Concern, which was declared on 5 May 2023 (WHO Director-General’s opening remarks at the media briefing – 5 May 2023). The aim was to compare SARS-CoV-2 RNA concentration dynamics in municipal wastewater with 15 regional public health indicators of diverse nature. We evaluated the effectiveness of the WS approach in describing the spread of SARS-CoV-2 across different epidemiological and surveillance periods. We also test the capability of WS for early warning by leading correlation. The periods before and after the Public Health Emergency (named ‘pandemic’ and ‘post-pandemic’, respectively) were compared as well as the periods before and after 50% of the French population received a vaccine dose. The circulation of variants of concern such as Omicron was also considered.

## 2 Materials and methods

### 2.1 Municipal WWTP settings and sampling strategy

The Montpellier metropolitan WWTP (Maera) collects both municipal and industrial wastewaters and stormwater from Montpellier and 18 neighboring towns representing 450,000 inhabitants which correspond to 37.3% of the total population of Hérault department (France) in 2020 (https://www.insee.fr). The sewer system primarily operates as a gravity-based and separate sewer network; the remaining combined sewage part (7%) is found within the historic city center. The flow of reference of the WWTP was 130,000 m3/day. The collection of flow weighted 24-h composite wastewater samples were carried out by the regulatory autosampler of the WWTP, in the inlet: once a week from 14 July 2020 to 11 January 2021 and from 2 May 2022 to 13 November 2025, and twice a week from 18 January 2021 to 25 April 2022. 125 mL samples were collected in sterile plastic bottles, transported in coolers to the laboratory and stored at 4 °C for processing within 2 h. The sampling and storage conditions did not change over the 5.5-year period. As stipulated in the 15 July 2015 decree^1^ the flow rate at the entrance of the WWTP is measured daily.

### 2.2 Wastewater sample preparation and RNA extraction

Two slightly different RNA extractions protocols were used: extraction protocol 1 (EP1) for samples collected from 14 July 2020 to 13 February 2023, and extraction protocol 2 (EP2) for all samples collected after this date. For both protocols, homogenized raw sewage (30 mL) was vortexed three times for 10 s each. In EP1, vortexed sample was centrifuged at 4 °C and 1,500 *g* during 10 min to remove most bacterial cells and debris and 15 mL of the resulting supernatant were subjected to ultrafiltration using Amicon® Ultra-15 10 kDa centrifugal filter unit (Millipore) via centrifugation at 4 °C and 3,234 x g for 35 min. In EP2, 15 mL of the vortexed samples was directly subjected to ultrafiltration following the same method. Finally, 140 μL or 150 μL (EP1 and EP2 respectively) of the concentrate was subjected to RNA extraction using the QIAamp Viral RNA Mini Kit (Qiagen) in EP1 or the NucleoSpin RNA Virus Kit (Macherey-Nagel, Germany) in EP2, according to the manufacturer’s instructions. The Pepper mild mottle virus (PMMoV) concentrations were used as quality control to compare EP1 and EP2 (Supplementary Figure 1). RNA extracts (60 μL) were stored at −80 °C until analysis.

### 2.3 Reverse transcription-digital-PCR

Absolute quantification of SARS-CoV-2 was made by IAGE company (Montpellier, France), using a TaqMan-based reverse transcription digital PCR (RT-dPCR). Primers and probes used for the quantification of SARS-CoV-2 RdRp gene (IP4 target) were designed by Institut Pasteur (France) (Pezzi et al., 2020). Mutations that could impact primers and probes hybridizations have been checked throughout the study^2^. The Pepper mild mottle virus (PMMoV) was also quantified (Symonds et al., 2018). The Limit of Detection (LoD) of SARS-CoV-2 primers and probes set was determined to be 10 copies per RT-dPCR reaction. The Limit of Quantification (LoQ) of both SARS-CoV-2 and PMMoV primers and probes set were determined to be 100 copies per RT-dPCR reaction. Optimization of the volume of RNA extract used in the RT-dPCR reaction was performed to reduce the impact of inhibitors on the polymerization step. In addition, an internal inhibition control (random RNA sequence) was added to each RT-dPCR assay to assess the presence of inhibition of each extract. RT-dPCR reactions were conducted using a multiplex assay developed by IAGE on a QIAcuity Eight Platform System (QIAGEN, Germany), using the QIAcuity One-Step Viral RT-dPCR Kit (Cat. No. 1123145) during the first part of the study and replaced by the actual QIAcuity OneStep Advanced Probe Kit (Cat. No. 250132). The RT-dPCR reaction mixtures were prepared in a plate as follows: for each reaction 10 μL of 4x OneStep Viral RT-dPCR Master Mix (or 4x OneStep Advanced Probe Master Mix), 0.4 μL of 100x Multiplex Reverse Transcription Mix (or 100x OneStep Advanced RT Mix), 5 μL of the primers/probes mix (2.25 μM of each primers, 0.625 μM of probe), and 4 μL of RNA extract were diluted with nuclease-free water to final volume of 40 μL. The reaction mixtures were transferred into a QIAcuity Nanoplate 26 K 24-well (Cat. No. 250001) and the plate was loaded onto the QIAcuity Eight automated system. The workflow included (i) a priming and rolling step to generate and isolate the chamber partitions (26,000 partitions), (ii) an amplification step with the following cycling protocol: a reverse transcription step at 50 °C for 40 min, 95 °C for 2 min for enzyme activation, 40 cycles with 95 °C for 5 s for denaturation, and 58 °C for 60 s for annealing/extension; and (iii) imaging step by reading fluorescence emission after excitation of the probe at the appropriate wavelengths. Results validation began with the verification of the number of valid partitions, which had to exceed 20,000 according to the manufacturer’s recommendations. Several controls were included in each RT-dPCR run: a negative dPCR control (molecular-grade water), a negative extraction control (extraction blank), an inhibition control and several positive controls (including both low- and high-concentration positive controls). Each run was considered valid when all quality controls produced the expected results. In cases where inhibition was monitored (i.e., inhibition control not detected in a sample extract), the assay was repeated using a diluted extract to alleviate inhibition. The low-concentration positive quality control, containing 30 copies of SARS-CoV-2 per reaction (corresponding to 3 × the LoD) ensured that samples in which SARS-CoV-2 was not detected could be considered true negative for this target. Data were analyzed using the QIAcuity Software suite V1.2. Results were given in gene copy number per microliter of RNA and transformed in gene copy number per liter of raw sewage (SARS-CoV-2 Genome Units/L, GU/L) as follows:


$$\begin{array}{l} \left(\frac{\begin{array}{l} \mathrm{GC}\;\mathrm{per}\;\mathrm{\mu L}\;\mathrm{RNA}\;x\;\text{total volume of extracted}\;\mathrm{RNA}\;(60\;\mathrm{\mu L})\;\times \\ \;\text{total volume of concentrated}\;\mathrm{WW}\;(\mathrm{\mu L}) \end{array}}{\begin{array}{l} \text{volume of concentrated}\;\mathrm{WW}\;\text{used for}\;\\ \mathrm{RNA}\;\text{extraction}\;(140\;\text{or}\;150\;\mathrm{\mu L}) \end{array}}\right)\\ \times (\frac{1000\;\mathrm{mL}}{\text{volume of}\;\mathrm{WW}\;\text{used for concentration}\;(15\;\mathrm{mL})}) \end{array}$$

Then concentrations of SARS-CoV-2 in GU/L were multiplied by the daily flow rate at the inlet of the WWTP to obtaine SARS-CoV-2 loads in GU/day. Since April 2021, PMMoV, considered as a constant baseline of human fecal load (Symonds et al., 2018), was used as quality control for comparison of two extraction protocols, for the standardization of SARS-CoV-2 quantification and for comparison with flow rate for SARS-CoV-2 load normalization. PMMoV quantification was expected to exceed 10^7^ GU/L in tested sample to validate the extraction process, based on laboratory expertise. Standardized quantifications were expressed by the ratio SARS-CoV-2 GU/L on PMMoV GU/L. The RT-dPCR conditions did not change over the 5.5-years period.

### 2.4 Public-health data and study periods

The city of Montpellier is in the Hérault department, which belongs to the Occitanie region. Over the pandemic period, from 14 July 2020 to 30 June 2023, the results of all screening and diagnosis SARS-CoV-2 tests performed in France were transmitted to the national information system named SI-DEP. From tests deposited in SI-DEP database, four SARS-CoV-2-specific indicators were calculated: the number and rate of positive tests, the incidence for 100,000 inhabitants and the effective reproduction number (Re). Also based on positive SARS-CoV-2 tests, indicators of medical activities linked to COVID-19 patients were calculated: the rate of intensive care rooms occupation, and the incidences of hospitalization, hospitalization in intensive care units and death for COVID-19 positives patients in Hérault. Over the study period (2020 to 2025), three non-specific indicators issued from syndromic surveillance without virological confirmation were available for Hérault department: medical and emergency procedures, and post-emergency hospitalizations. All departmental indicators were collected from open data portal of Santé Publique France^3^.

From 1 January 2023 to 13 November 2025, four indicators were calculated for Montpellier hospital from SARS-CoV-2 laboratory and hospitalization data: the number and rate of positive tests performed in the virology laboratory of the hospital of Montpellier, as well as the numbers of patients being admitted to hospital due to severe COVID-19 (named “patients for COVID-19”) or being admitted for other reasons and tested positive for SARS-CoV-2 upon admission (named “patients with COVID-19”).

Data analyses were carried out by period according to the availability of each public health indicator (Supplementary Table 1). The SARS-CoV-2 epidemic waves were assigned based on the official declaration of waves in France, adapted slightly to have no gap between waves (Table 1). As the official 8th wave was very short, we have grouped data concerning official 8th and 9th waves under the name Wave 8 in this study. Finally, the data analyses considered periods before and after events that altered the dynamics of the virus or the disease, such as vaccination, the emergence of Omicron variants, and changes in disease management, from crisis (pandemic) to routine (post-pandemic) (Table 1 and Supplementary Table 1).

**Table 1:** Synthesis of Spearman correlation analyses. Spearman correlation coefficients (*r_s_*) calculated on actual data between SARS-CoV-2 load in wastewater and 15 public health indicators for different periods and epidemic waves.

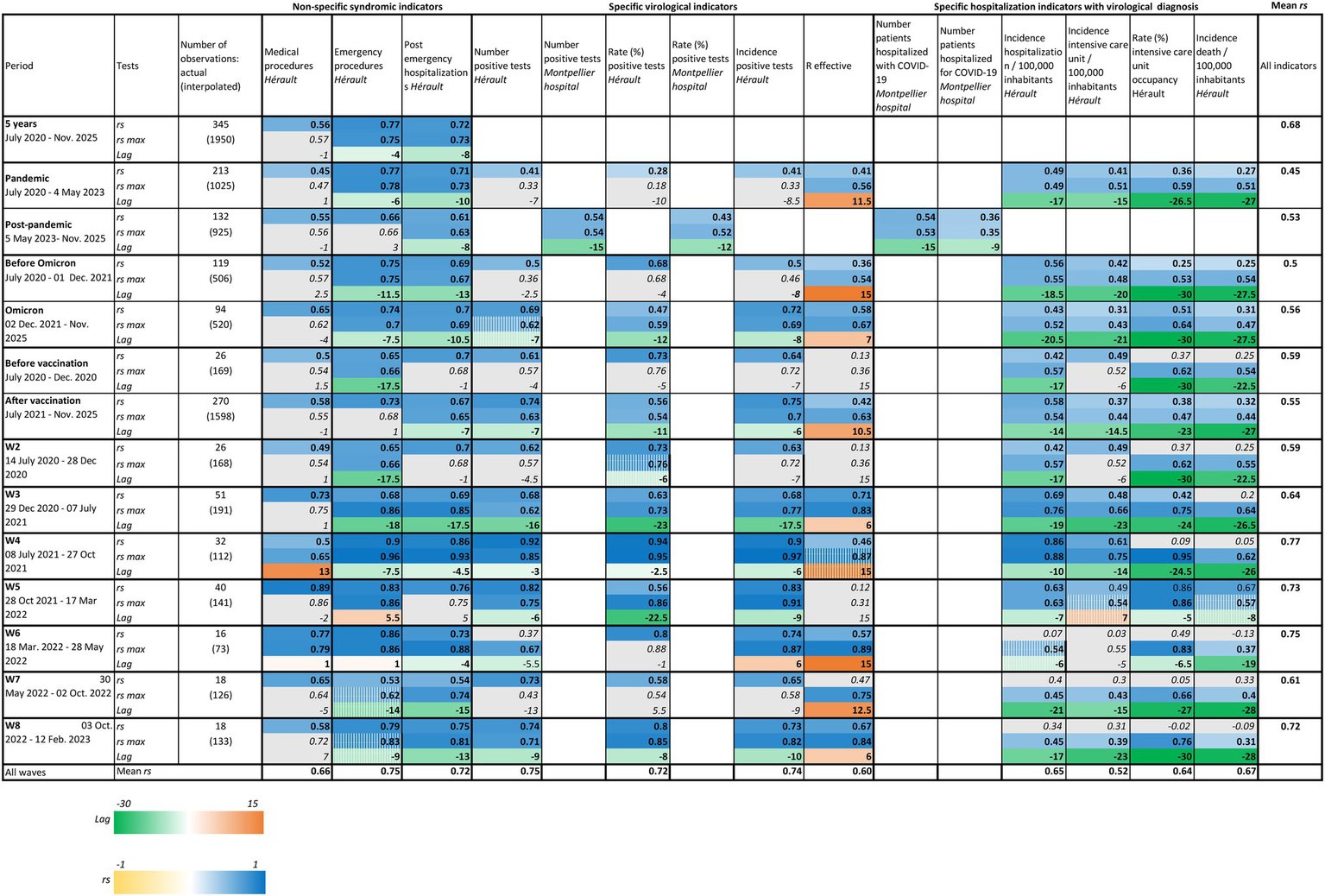

Maximum Spearman correlation coefficients (*r_s_* max) determined on daily-interpolated data by shifting wastewater data by one day over a 30-day period before, and by one day over a 15-day period after each public health indicators. *Lag* corresponded to the shift in day giving *r_s_* max. White boxes when public-health indicators are not available. The coloured boxes indicate a significant rs (*p*-values ≤0.05, corrected by Benjamin-Hochberg procedure) or a significant rs max validated by the bootstap IC and a bootstrap *p*-value ≤0.05 as described in the materiel and methods section. All these significant values are in bold. The coloured hatched boxes indicate a rs max not significative according to the bootstrap IC but with a bootstrap *p*-value ≤0.05. Non-significant rs, rs max and Lag (p-value > 0.05) are indicated in light gray and italic. The heatmap colour legend is at the bottom of the table. The green to orange scale shows the value of the lags and the yellow to blue scale shows rs and *rs* max.

### 2.5 Statistics

The normality of data was tested using a Shapiro–Wilk test. The comparison of SARS-CoV-2 concentration and loads, PMMoV concentration and SARS-CoV-2/PMMoV ratio according to epidemics waves or events were done by Mann–Whitney and Wilcoxon test. To evaluate the correlations between variables that do not follow a normal distribution, Spearman correlation coefficients (*r_s_*) were calculated. Due to the large number of correlation tests on the same dataset, the procedure of False Discovery Rate (Benjamini-Hochberg) was used to control the proportion of false positives among significant results. Unlagged correlation has been assessed from actual (non-interpolated) data. The na.spline function from the R package “zoo” was used to interpolate daily data between actual results from wastewater analyses, enabling comparisons to be made between weekly or twice weekly SARS-CoV-2 concentrations in wastewater and the various daily health indicators. Likewise, R_e_, the number of medical and emergency procedures, and post-emergency hospitalization in the department for COVID-19 data were interpolated from the actual weekly data. To assess maximum correlation and lagged association, the interpolated wastewater data were shifted by one day over a 30-day period before, and by one day over a 15-day period after, the public health indicators. When the wastewater data preceded the public health indicators, the shift was indicated as negative. Thus, a negative lag indicates wastewater leads the clinical indicator by a number of days corresponding to the lag. The significance of the shift that led to maximum correlation was tested using the moving block bootstrap method by the *tsboot* function from the boot package, in order to take into account autocorrelation and data dependency due to interpolation. Block lengths were estimated as follows: for each indicator and temporal analyses, data were simulated with varying size block (ranging from 5 to 30 in increments of 2) in a shorter bootstrap (200 repetitions). Then, the block length which minimizes the mean squared error was selected. The bootstrap produced new correlation results (bootstrap r) for a Lag x during 1,000 replications and 95% confidence intervals (CI) were calculated. The null hypothesis—that the correlation is due to random chance—was rejected if the r calculated from the original dataset fell outside the bootstrap-generated confidence interval. The corresponding *p*-value was the probability that correlation from bootstrap resampling was higher than the maximal correlation from original non-bootstrapped data.

All results were significant for p-value ≤ 0.05. All analyses used pairwise complete observations based on the availability of observation as detailed in Supplementary Table 1. The categories of correlation were defined as follows: 0.00–0.29, Weak; 0.30–0.49, Moderate; 0.50–0.75, Strong; > 0.75, very strong. All statistics were done with R version V4.5.1 with RStudio interface version 2026.01.0 + 392.

## 3 Results

### 3.1 Five-year dynamics of SARS-CoV-2 RNA in municipal wastewater and individual tests

In response to the first wave of COVID-19 pandemic, the French government declared the state of emergency and imposed the population lockdown on 17 March 2020. The strict lockdown period ended on 11 May 2020 but restriction for travel, schools and access to public places was maintained until 2 June 2020. The study began on 14 July 2020 in the early post-lockdown period and was pursued up to 13 November 2025. The critical dates used to define the epidemiologically-relevant periods were given in Table 1 and Supplementary Table 1.

**Figure 1:**
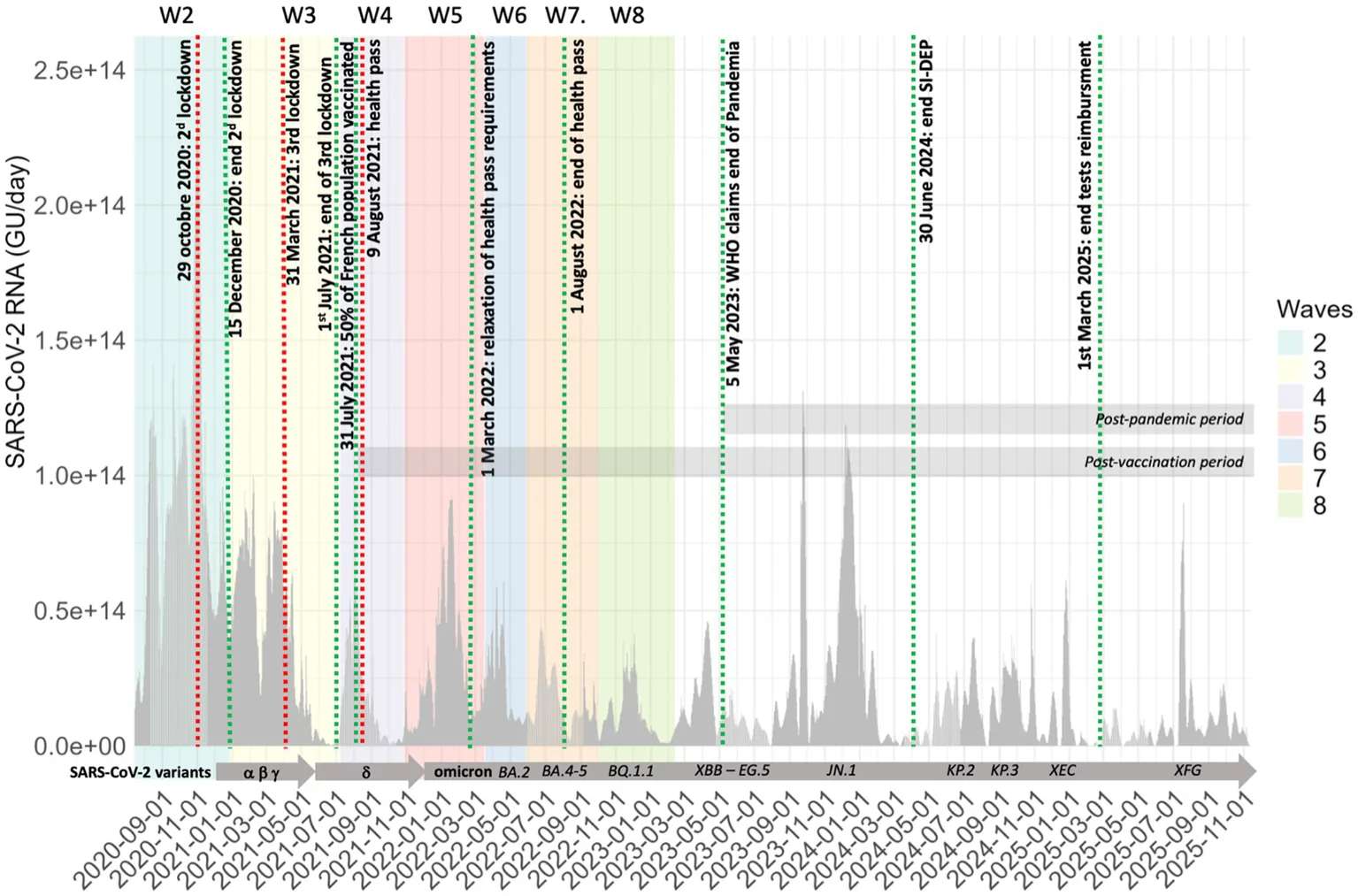
Dynamics of SARS-CoV-2 IP4 gene expressed in genome units per day (GU/Day) during the whole study period. The different epidemic waves or period were identified by different background colors (see the caption) and are indicated by a W followed by the number of the wave. The dominant SARS-CoV-2 variants are indicated by grey arrows at the bottom of the graph according to public data (https://odisse.santepubliquefrance.fr). Several landmarks corresponding to French or World Health Organization policies are indicated by dotted lines: red for public health constraints, green for measures relaxation. Post-pandemic period (after World Health Organization claims the end of pandemic) and post-vaccination period (when 50% of the French population received complete primo vaccination) were indicated by grey boxes.

The temporal dynamics of SARS-CoV-2 RNA over 65 months (5.5 years) are reported to the daily flow rate at the WWTP and expressed in genome units per day (GU/day) for a total of 345 samples of wastewater (Figure 1). SARS-CoV-2 concentration (GU/L) and SARS-CoV-2 load normalized by daily flow (GU/day) were very strongly correlated (*r_s_* = 0.98; *p*-value<0.001). To validate the normalization of SARS-CoV-2 RNA concentration by flow rate, the concentration of PMMoV and the SARS-CoV-2/PMMoV ratio were measured in each sample from April 2021 until the end of the study. The dynamics of the SARS-CoV-2/PMMoV ratio were highly correlated with SARS-CoV-2 load in GU/day (*r_s_* = 0.91; *p* < 0.001) (Supplementary Figure 2), thus validating the flow rate-based normalization. In case of significant rainfall, flow normalization may not fully correct dilution or signal loss. This could occur when the WWTP’s reference flow is exceeded. The reference flow was exceeded in 15 out of 345 observations (4%). Of these, only one was SARS-CoV-2-negative, which was consistent with samples taken during the same period that showed low or negative SARS-CoV-2 signals (data not shown). In cases of overflow, some of the SARS-CoV-2 signal may be lost, particularly if the overflow affects a sub-catchment with a high prevalence rate. Under the same conditions, the PMMoV signal does not decrease because the concentration is considered population-dependent and therefore homogeneous in the various sub-catchments. No anomalies were detected between flow and PMMoV normalizations for these 15 observations (data not shown).

SARS-CoV-2 RNA signal was always detected except in 11 samples collected from March 2024 to November 2025. During the study, concentrations ranged from 2.84 × 10^3^ to 3.02 × 10^6^ IP4 genes copies /L or GU/L of raw sewage corresponding to 6.16 × 10^10^ to 2.41 × 10^14^ GU/day (Figure 1). The second wave in France—the first monitored in this study—was the highest observed, reaching a maximum of 2.41 × 10^14^ GU/day on 7 November 2020. It was also the longest, lasting approximately 5 months from July to December 2020. A second increase occurred from December 2020 onwards, with a load reaching approximately 1 × 10^14^ GU/day on 2 February 2021. This resulted in a double-peaked curve corresponding to the third wave in France (Figure 1). The second and third national lockdowns were linked to sudden decreases in wastewater SARS-CoV-2 load followed by a rapid increase just before the end of lockdowns. The wastewater peak corresponding to the fourth wave (July to October 2021) was lower. The fifth wave (November 2021 to March 2022, the first wave involving the variant Omicron) reached a level like that of the third peak, but over a shorter period. The four subsequent peaks in 2022 and 2023 became less pronounced, until the post-pandemic period which was marked by high but brief peaks > 1 × 10^14^ GU/day (end September and mid-December 2023), the period of emergence of Omicron JN.1 in France. In 2024–2025, SARS-CoV-2 signals in wastewater were irregular, with relatively low levels interspersed with sporadic, short-lived peaks lasting 1–2 weeks (Figure 1).

**Figure 2:**
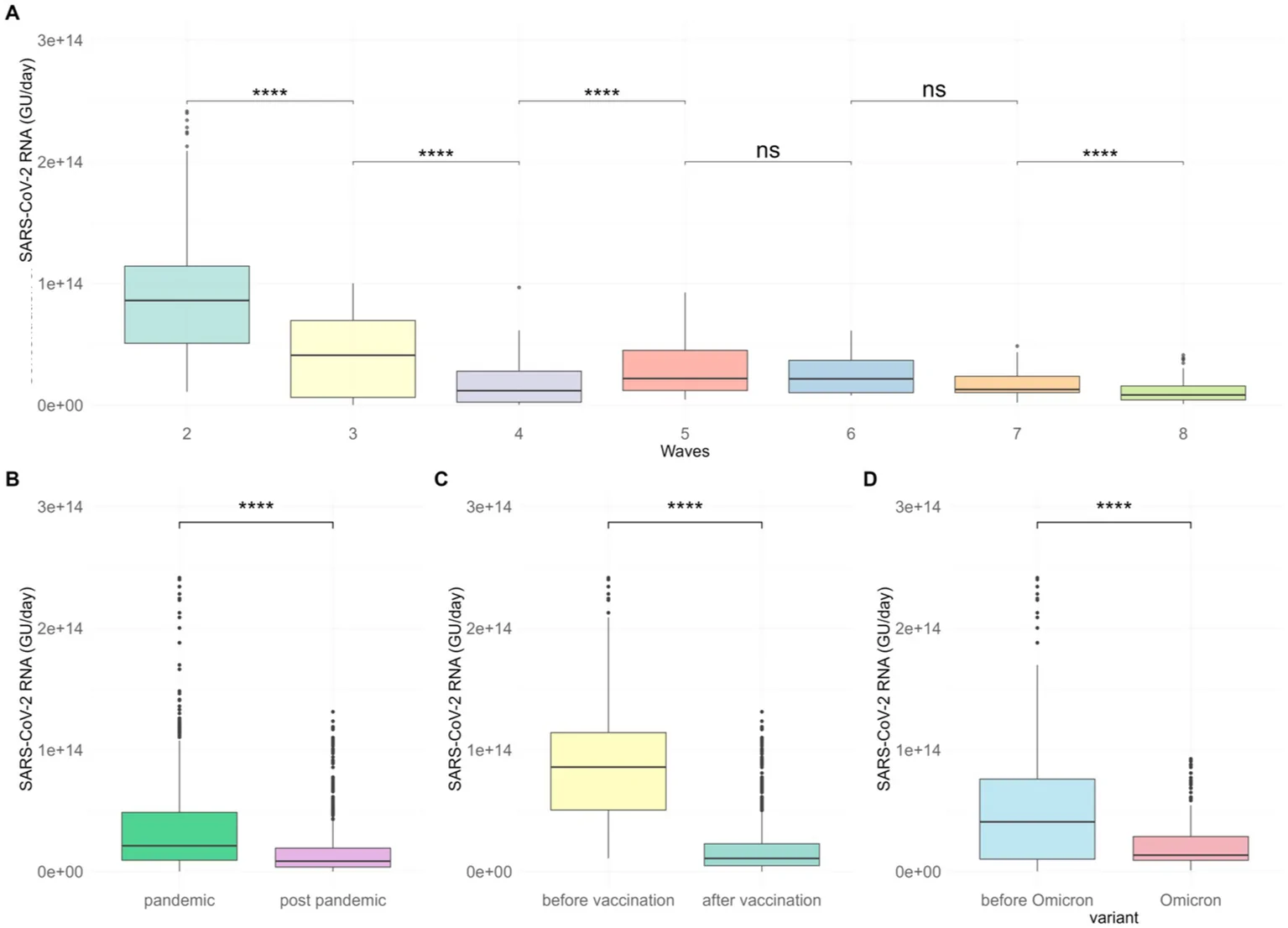
Distribution of SARS-CoV-2 RNA load (GU/Day) according to **(A)** epidemic waves; **(B)** pandemic and post-pandemic periods; **(C)** before and after Omicron emergence in the pandemic period and **(D)** before and after primo-vaccination of 50% of the population in the pandemic period. Comparison were performed by U Mann–Whitney test. Significance is indicated by **** corresponding to a *p*-value ≤ 0.0001 or by NS for non-significant.

The Figure 2 shows the comparison of SARS-CoV-2 RNA loads distributions across waves and periods. Over the study period, a trend towards lower signal levels was observed, with median SARS-CoV-2 RNA loads decreasing from 8.6 × 10^13^ to 7.48 × 10^12^ GU/Day (Figure 2A). The signal decreased mainly between waves 2 (original wild-type virus), 3 (*α*, *β*, *γ* variants) and 4 (*δ* variant) before a partial increase associated with Omicron variant in wave 5. During the Omicron period, the median SARS-CoV-2 load in wastewater decreased. The decrease of wastewater signal was significant between pandemic and post-pandemic periods (*p*-value < 0.001) (Figures 1, 2B). The effect of variants and vaccination on the wastewater signal were tested only during the pandemic period (waves 2 to 8) to avoid biases linked to the decrease of signal between pandemic and post-pandemic. Significantly lower loads of SARS-CoV-2 RNA in wastewater was observed for the period of the Omicron variant circulation compared to the period covering the circulation of wild-type, Alpha, Beta, Gamma and Delta variants (Figures 1, 2C) (*p*-value < 0.001). The vaccination status of the population appeared associated to lower loads of SARS-CoV-2 RNA in wastewater with a significant decrease in wastewater signal after 50% of the French population had received the first vaccine dose at the end of June 2021 (*p*-value < 0.001) (Figures 1, 2D).

**Figure 3:**
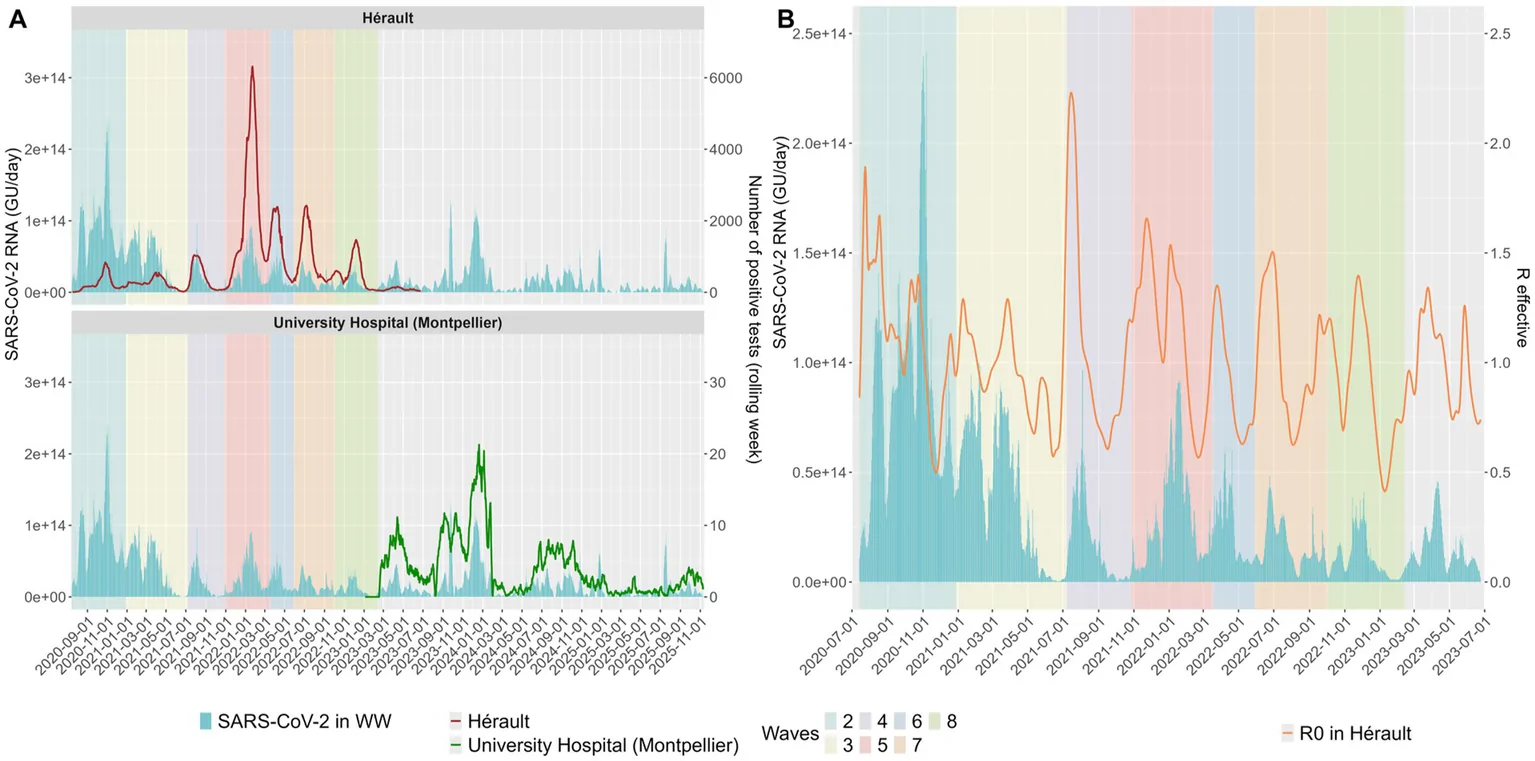
Dynamics of SARS-CoV-2 in GU/Day during the 65 months of the study period compared to **(A)** the number of positive individual tests in Hérault (red curve, before 1 June 2023) and the number of positive individual tests in the regional hospital (green curve, after 1 January 2023); **(B)** the R effective (Re) in Hérault during 3 years of the pandemic period. The different epidemic waves were represented by different background colors (see the caption on the figure).

The peaks of SARS-CoV-2 RNA loads in municipal wastewater appeared concomitant with the peaks of positive individual tests in Hérault (red curve in Figure 3A), themselves linked to the epidemic waves that occurred in France during the pandemic period, except for the first wave not included in this study. Similar results were obtained with the rate of positive tests (Supplementary Figure 3). Comparison between SARS-CoV-2 loads in wastewater and the effective reproduction number (R effective, R_e_) showed very similar dynamics over the pandemic period (Figure 3B). This suggested that SARS-CoV-2 load in wastewater reflected not only the fecal viral excretion but also the transmission rate in the population.

Positive tests data were unavailable in Hérault in the post-pandemic period, because the SI-DEP system ceased to function on 30 June 2023. From this date onward, positive tests performed at the regional hospital were used as individual virological indicator instead of positive tests in Hérault (green curve in Figure 3A). The comparison of these two indicators when they were both available, from January to July 2023, showed very strong correlation: *r_s_* = 0.799 for the number of positive tests (Supplementary Figure 4) and *r_s_* = 0.81 for the rate of positive tests (data not shown), *p*-values < 0.001. This strong correlation may be explained by the fact that the hospital of Montpellier carried out more than half of the SARS-CoV-2 tests in the metropolitan area of Montpellier and because the hospital tests were carried out patients hospitalized with COVID-19, which better reflect the community population than patients hospitalized for severe COVID-19.

From the fifth wave (first Omicron wave), the positive tests number was relatively high compared to wastewater signals, unlike the earlier waves (Figure 3A and Supplementary Figure 3). This was likely due to changes in testing policies in France, such as the end of the health pass. Tests were then primarily performed on symptomatic patients, resulting in fewer tests but a higher positive rate. The absolute number of positive tests and the incidence of positive tests per 100,000 inhabitants, compared to wastewater signals, showed reduced peaks for waves 6 and 7, but not for wave 5 (Supplementary Figure 5). Thus, for wave 5, the difference in height between positive tests and wastewater signals probably reflected lower SARS-CoV-2 RNA load in wastewater despite a high population incidence. Notably, a high peak in positive tests occurred in late July 2025 during the post-pandemic period without a corresponding increase in wastewater signals (Figure 3A). These latter observations suggested variation in SARS-CoV-2 excretion at the population level.

### 3.2 Correlation between SARS-CoV-2 RNA load in wastewater, individual testing and public health indicators

**Figure 4:**
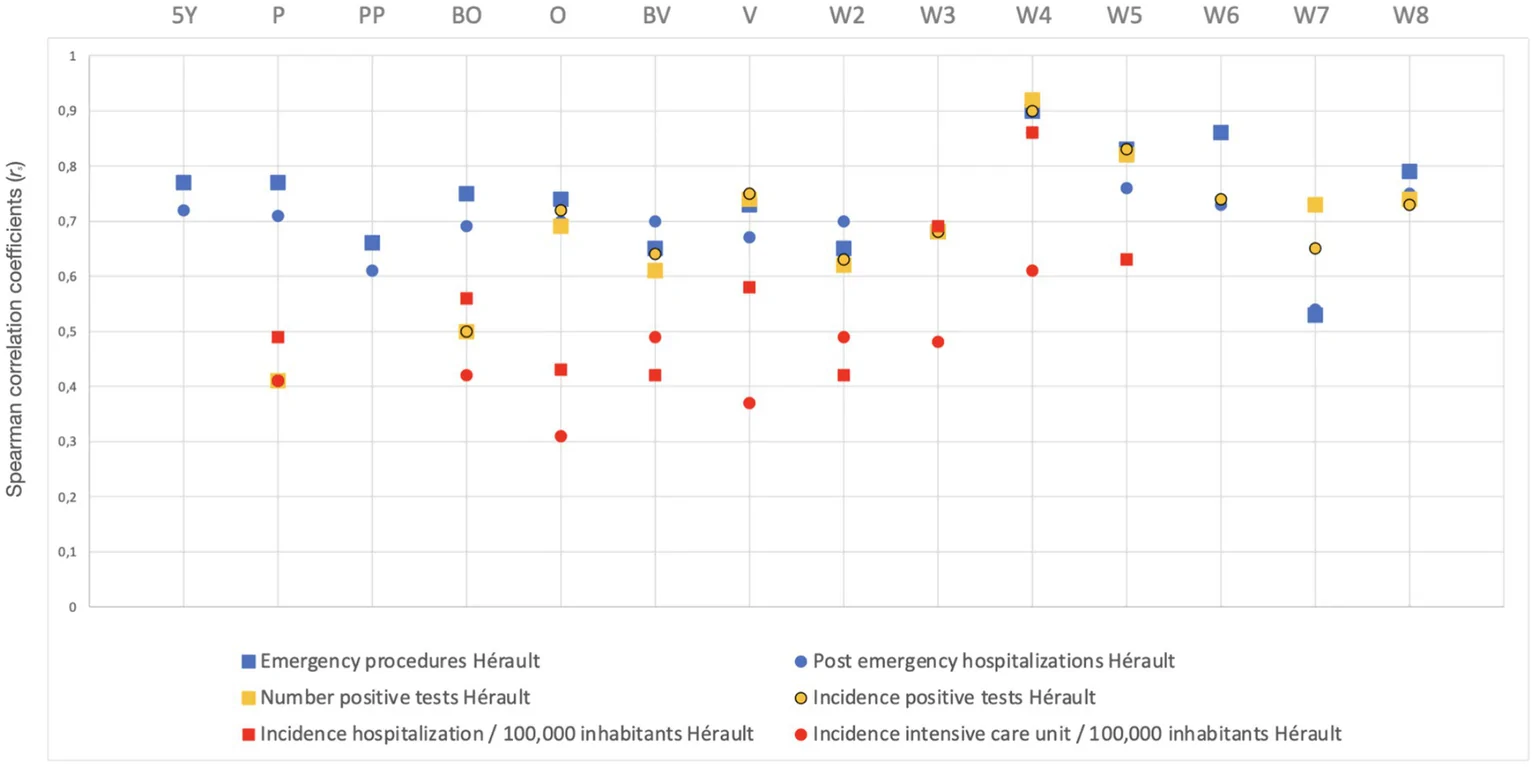
Distribution of Spearman’s correlation coefficient (*r_s_*) between SARS-CoV-2 RNA load in GU/Day and 6 public-health indicators: non-specific syndromic indicators in blue (*n* = 2), specific virological indicators in yellow (*n* = 2) and specific hospital indicators in red (*n* = 2) at different time periods. 5Y for 5.5-years period; P for pandemic period; PP for post-pandemic period; BO for before Omicron; O for Omicron; BV for before vaccination; V for after vaccination; W1 to W8 for the corresponding epidemic waves.

The *r_s_* adjusted with False Discovery Rate procedure were calculated to evaluate the correlation between SARS-CoV-2 RNA load in wastewater and public health indicators. Twelve specific indicators based either on virological individual tests or confirmed diagnoses of COVID-19 and 3 non-specific syndromic indicators (independent of specific virological tests) were considered, according to their availability throughout the study (Supplementary Table 1). For clarity, Figure 4 shows the strongest correlations for four specific, and two non-specific indicators, while Table 1 lists *r_s_* values for the 15 indicators across periods and epidemic waves.

Over the 5 years of the study, the number of medical and emergency procedures and the post-emergency hospitalizations showed strong and very strong correlations with wastewater data (*r_s_* = 0.56, 0.77 and 0.72 respectively; *p* < 0.05). During the pandemic, the *r_s_* values for these three non-specific indicators were consistently higher than those for virological indicators (Figure 4 and Table 1). Correlations with emergency procedures, and post-emergency hospitalizations remained high during the post-pandemic period (*r_s_* = 0.66 and 0.61, respectively; *p* < 0.05).

Despite globally similar dynamics (Figure 3), wastewater SARS-CoV-2 RNA loads showed a weak correlation with the rate of positive individual tests in the Hérault region during the pandemic period (*r_s_* = 0.28; *p* < 0.05), and a moderate correlation with the absolute number of positive individual tests, the incidence rate per 100,000 inhabitants and the R effective (*r_s_* = 0.41 for all; *p* < 0.05) (Figure 4 and Table 1). Moreover, the number and the rate of positive tests in Montpellier hospital were strongly (*r_s_* = 0.54; *p* < 0.05) and moderately (*r_s_* = 0.43; *p* < 0.05) correlated with SARS-CoV-2 load in wastewater during the post-pandemic. Hospitalization and mortality indicators based on virological diagnosis were moderately to highly correlated with wastewater signals with lower correlation coefficient than non-specific indicators (Figure 4 and Table 1). Correlations of every indicator with the load of SARS-CoV-2 in wastewater were also calculated for epidemiologically significant periods in pandemics and for each epidemic wave (Figure 4 and Table 1). The *r_s_* of 3 indicators directly derived from positive tests (number, rate and incidence) – which are affected by the total number of tests realized - varied independently across periods. However, at least one of them was very strongly correlated with wastewater signal during every epidemic wave. Comparison of *r_s_* before/after vaccination or before Omicron/Omicron periods were performed (Table 1). The correlation category changed between these periods for most indicators but with a maximum of one step. Most *r_s_* were strong or very strong indicating good association of SARS-CoV-2 in wastewater with public-health indicators whatever the epidemiological period (Figure 4 and Table 1). Finally, the mean correlation coefficient calculated for each indicator across all epidemic waves (Table 1) indicated that the emergency procedure (mean *rs* = 0.75), post-emergency hospitalizations (mean *rs* = 0.72), the number and incidence of positive tests in Hérault (mean *rs* = 0.75 for both) and the rate of positive tests (mean *rs* = 0.72) displayed the strongest correlation with SARS-CoV-2 load in wastewater.

### 3.3 Testing the early warning of SARS-CoV-2 RNA in municipal wastewater using lag-association with public health indicators

**Figure 5:**
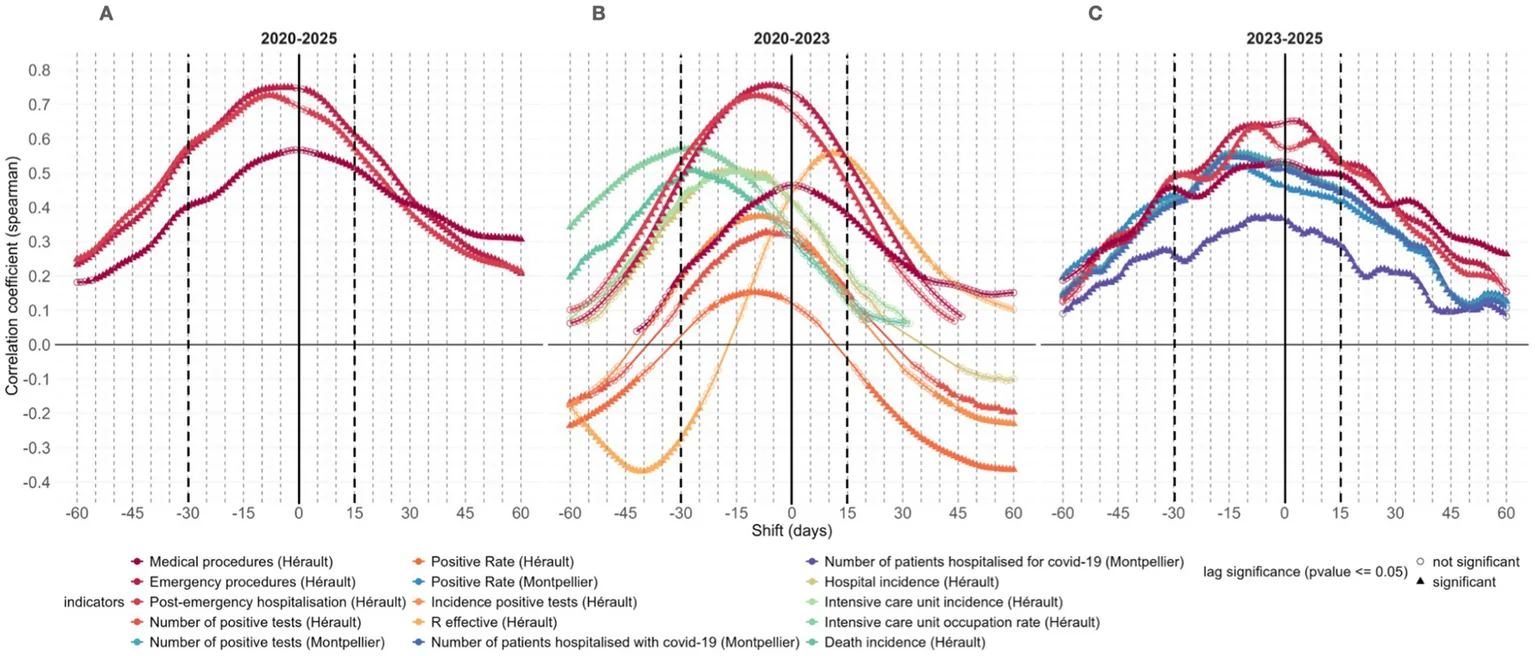
Evolution of Spearman correlation (*r_s_*) coefficient according to day shift. Correlation coefficients are calculated on daily interpolated data between SARS-CoV-2 in GU/Day load in wastewater and the health indicators measured for 3 periods **(A)** 2020–2025, **(B)** the pandemic period (2020–2023), **(C)** the post-pandemic period (2023–2025). Lags were considered significant if the bootstrap *p*-values for rs were ≤0.05. The same analysis performed for each wave is presented in Supplementary Figure 6.

To evaluate the leads or the lags between SARS-CoV-2 RNA in wastewater and public health indicators, lag-association tests were performed 30 days before and 15 after every day of monitoring. This range accounts for the infection’s known progression, including the delay between exposure and ICU admission for severe cases (~30 days), while avoiding overlap with the previous epidemic wave (≤15 days prior). The load of SARS-CoV-2 RNA in wastewater on a given day were shifted by negative and positive increments of one day and then compared to the 15 public health indicators. The evolution of *r_s_* according to the day shift is given in Figure 5 for 3 periods depending on the availability of each indicator. The shift yielding the maximum *r_s_* (*r_s_* max) is listed in Table 1.

After bootstrap analysis, 26.7% of shifts giving maximum correlation were statistically non-significant (light gray in Table 1) based on both confidence interval and *p*-value (32.4%) or only confidence interval (5.7%) (hatched in Table 1). This is particularly the case for medical procedures with syndromic diagnosis and for early pandemic periods (before Omicron and before vaccination, wave 2) but also for later waves such as 7 (Table 1). This may be due to the rapid succession of peaks and variants during the early pandemic, resulting to heterogeneous epidemiological waves. When significant, maximum correlations between public health indicators and wastewater signals were obtained for negative lags in 83% of the tests (Table 1 and Figure 5). For most indicators, the difference between *r_s_* and *r_s_* max was low but higher for COVID-19-related hospitalization and mortality indicators, for which the correlation coefficient shifted from non-significant to significant and high or very high. This shows that the increase in wastewater signals generally preceded the increase in public health indicators. Wastewater signals led public health indicators by 2.5–30 days, depending on the period and wave (Table 1). WS predicted virological indicators (except R effective) with an average lead time of 10 ± 7 days, and hospital specific indicators (except death) with a lead time of 19 ± 7. For 13.5% of the significant tests, a positive lag is observed, i.e., public health indicators led wastewater signal (positive lags). This was particularly true for R effective, which generally preceded wastewater signals (positive lags). This may be because R effective is calculated *a posteriori* using data from the previous week (Figure 5B).

There were no obvious differences in early warning potential of SARS-CoV-2 WS between the periods before and after the end of the pandemic, the Omicron variant’s spread, and vaccination coverage. By contrast, the effect of the shifts between wastewater data and public health indicators on correlations varies greatly according to the wave (Table 1 and Supplementary Figure 6). Notably, the Wave 5 (the first Omicron wave) in wastewater predicted the rate and incidence of positive tests, but non-specific indicators increased in advance compared to wastewater signal, despite the high correlation coefficients (Table 1). It is noteworthy that significant negative lags were observed for specific indicators based on individual virological tests in Hérault (orange shades in Figure 5B) and in Montpellier Hospital (turquoise shades in Figure 5C). This indicates that wastewater signals preceded individual virological tests, which are the main indicators used to monitor the pandemic.

Finally, the chronology of maximum correlation (Figure 5) indicates that non-specific syndromic indicators preceded specific virological indicators, which in turn preceded specific indicators for hospitalization and death. As expected, wastewater signal was earliest for indicators which measured late events in the medical course of patients.

## 4 Discussion

The monitoring of viral nucleic acids in wastewater as a proxy of human epidemiology has come into its own thanks to the COVID-19 pandemic. An increasing number of publications demonstrate correlation between SARS-CoV-2 RNA in wastewater and clinical incidence of COVID-19 (Parkins et al., 2024). However, the meta-analysis by Li et al. (2023) temperated these conclusions with only 12 publications showing significant correlation among the 27 selected for meta-analysis. The authors highlighted the importance of viral shedding dynamics relative to the epidemic phase (e.g., pre- or post-peak of the outbreak) and the type of clinical testing in accurately estimating the number of cases of SARS-CoV-2 infection through wastewater monitoring (Li et al., 2023). More generally, associations between SARS-CoV-2 RNA concentrations in wastewater and clinical indicators should be interpreted with cautiously because WS provides an aggregated population-level signal reflecting collective viral shedding rather than individual infection events (Goetgeluck et al., 2025).

Our 5.5-year longitudinal study in Montpellier validates the overall correlation between wastewater signals and human epidemiology while demonstrating that this relationship fluctuates across different epidemiological periods and among the 15 public health indicators analyzed. The primary novelty of this study is that wastewater samples were analyzed over an extended period by the same laboratory. This allowed us to assess whether correlation persists under changing epidemiological and surveillance conditions – vaccination progression, variant turnover, reduced individual testing, endemic transmission, and altered hospital burden. Another strength is the study ability to compare wastewater signals with a wide range of public health indicators, although not all of these being available throughout the study period.

The study’s ambitious aims introduced some limitations, particularly regarding statistical significance and correlations. The wide number of tests due to the number of indicators and periods may generate Type I error requiring adjustments by False Discovery Rate procedure. Another issue is the sampling frequency feasible during a long-term period. Actual data were obtained only once or twice a week, depending on the period whereas two or three samples per week are now recommended by World Health Organization (2025). Then, a simulation was used to convert these into simulated daily data, which do not reflect actual variations and could increase autocorrelation. For analyses based on interpolated data, the significance was tested by block bootstrapping to limit data autocorrelation and dependency. Another challenge for this project was maintaining the same protocol throughout the 5.5-year study. Only the RNA extraction protocol was slightly modified in February 2023. The modified protocol yielded a 12% increase in the PMMoV signal in wastewater (Supplementary Figure 1). The data were not corrected because a 12% change in the wastewater signals did not affect the overall dynamics of SARS-CoV-2 in wastewater and the correlation results. In addition to unlagged correlations, the study aimed to evaluate WS prediction ability using lag association, which does not provide true forecasting. This is because the WS signal has only been shown to precede clinical indicators retrospectively, not prospectively. Real-time prospective forecasting is rare in the literature (Johnson et al., 2026), and future studies with specific designs are needed to explore WS’s actual forecasting potential. A key point of the WS methodology is normalization of signal (World Health Organization, 2022). In this study, flow-based normalization was selected, and its robustness was demonstrated compared to PMMoV normalization, even when extreme rainfall exceeded the WWTP’s reference flow. The Mediterranean rainfall regime in Montpellier leads to flash floods that are unlikely to introduce significant bias in long-term monitoring, but which may pose a challenge to real-time or short-term surveillance. Future WS studies should validate WS normalization in relation to extreme rainfall and more generally to various environmental variables (Siri et al., 2024).

Of the 15 indicators available during the study, 12 were specific - calculated from laboratory-confirmed SARS-CoV-2 infections or carriages in individuals - while 3 were non-specific, based on syndromic COVID-19 suspicion without virological confirmation. A higher level of correlation with virological indicators (number, rate, incidence of positive tests) was expected because they reflect the direct link between population-level viral shedding and viral presence in feces, urine, saliva and sputum (Cevik et al., 2021). The longitudinal study confirmed significant correlations between wastewater and virological indicators across most periods and waves. For instance, across all epidemic waves, we observed a high correlation (mean *r_s_* = 0.74) for the incidence of positive tests, which is a key indicator in the surveillance and alert systems during the pandemic. Correlations were weaker when longer periods, such as the entire pandemic period, were considered. This could be due to variations in virological screening and diagnostic strategies over time (Boehm et al., 2023) or in variant fecal shedding (Yang et al., 2025). A previous study (Rector et al., 2023) reported a smaller increase in SARS-CoV-2 RNA load in wastewater during the Omicron BA.1 wave than for previous variants of concern. The authors hypothesized that this was due to lower levels of fecal excretion of Omicron variants (Rector et al., 2023). Using wastewater data, Yang et al. (2025) modeled a decrease of fecal shedding for the Delta (decreased of 20%) and Omicron (decreased of 50–60%) variants. The dynamics observed in this study confirmed a decrease of wastewater signals in wave 4 (Delta variant) compared to R effective and during the Wave 5 (Omicron variant) compared to the number and rate of positive virological tests.

We observed a regular decrease in wastewater signal from the beginning to the end of the follow-up during the post-pandemic period. This was observed despite improved RNA extraction performance after February 2023. Indeed, during post-pandemic period, most of the population had developed immunity due to infections or vaccination, likely resulting in low-noise, endemic chain of transmission rather than outbreaks (Ulloa-Medina et al., 2026). However, the December 2023 post-pandemic wastewater peak - corresponding to the emergence of Omicron JN.1 in France (unpublished data from Santé Publique France) – exhibited a classic epidemic peak shape, with high correlations with most indicators. This suggests that WS can detect viral concentration changes correlating with individual test results within a population, even during low-noise, long-term endemic circulation of SARS-CoV-2. The variant JN.1 is described to evade population immunity (Kim et al., 2025). Furthermore, the detection of a high, fleeting SARS-CoV-2 peak in wastewater in July 2025 - without a corresponding increase in case-based virological indicators - may reveal a transient population-level increase in incidence undetected by the case-based surveillance system. Possible explanations include changes in shedding dynamics, hospital indicator insensitivity or - more likely - the lack of sufficient individual testing during the late post-pandemic period and summer holidays, with population movements. Moreover, stochastic noise at lower prevalence cannot be ruled out.

Positive individual test results were used to calculate indicators of hospitalization and mortality due to SARS-CoV-2 infections. These indicators were less significant and showed weaker correlation with wastewater signal than raw positive test indicators. This was particularly evident after Wave 5, likely due to an increase in asymptomatic or mildly symptomatic individuals shedding the virus. This likely decoupled SARS-CoV-2 excretion in wastewater from hospitalization indicators (Ulloa-Medina et al., 2026).

Throughout the entire period, non-specific indicators — based on syndromic diagnosis of COVID-19 without virological confirmation, such as emergency procedures and hospitalizations after emergency procedures — were also correlated with the load of SARS-CoV-2 in wastewater. However, syndromic indicators are influenced by influenza virus and various other respiratory pathogens. Their correlations were often the strongest, even though the other indicators were virologically confirmed and thus presumably more accurate. Such high correlations between wastewater signal and syndromic indicators were recently described in Germany (Abunijela et al., 2026). One possible explanation is that syndromic surveillance is well-established in France and Europe. For example, the emergency procedure data were sourced from the OSCOUR network, representing 96% of French emergency procedures. Thus, syndromic surveillance could be more accurate than specific surveillance due to its extensive coverage. Another hypothesis is that respiratory symptoms were almost exclusively caused by SARS-CoV-2 during the pandemic. In the post-pandemic period, other respiratory viruses recirculate and non-specific syndromic indicators become less correlated with SARS-CoV-2 in wastewater. Moreover, policy changes regarding virological testing led to increasing at-home testing and reduced willingness to undergo testing, with an increasing proportion of positive cases going unreported to public health authorities. This may obscure community epidemiological patterns (Rader et al., 2022). It may also explain why - during certain study periods- the wastewater signal was more closely correlated with syndromic indicators than with virological tests-based indicators. Based on similar observations, publication suggests that the viral signal in wastewater may provide a less biased assessment of community-level trends in the prevalence of SARS-CoV-2 (Strike et al., 2025).

Another advantage of monitoring SARS-CoV-2 in wastewater is that it could provide an early warning of epidemic waves (Doss et al., 2025; Cañas Cañas et al., 2025). In this study, shifts in the data that maximized the correlation coefficient were mostly negative. This suggests that changes in SARS-CoV-2 load in wastewater generally precede changes in public health indicators, thus confirming the early warning potential of SARS-CoV-2 WS. The main exception was R effective which generally preceded wastewater signals probably because it is determined *a posteriori*. However, the curves showing the dynamics of correlation coefficients according to data shifts were rather flat, leading in several cases to very small differences in the correlation coefficient over several consecutive days of shift and therefore non-significant results. As previously noted, an increase in frequency of observations as recommended by WHO could increase the forecasting power of WS. Nevertheless, during 2020–2023, when the greatest number of indicators were available, wastewater signals preceded the indicators as expected, based on their early or late stages in the SARS-CoV-2 infection progression and patient management.

Over the course of 5.5 years of SARS-CoV-2 WS, variations were observed in SARS-CoV-2 dynamics and variants, surveillance intensity, and population immunity. These factors may affect the virus load in wastewater or the accuracy of public health indicators. A key question was whether these differences could affect the correlation between wastewater and public health data. Correlations and predictive power tested before and after Omicron emergence, before and after 50% vaccination coverage, and before and after pandemic end declaration were roughly similar. These results suggested that a single method can be used throughout long-term WS of SARS-CoV-2 even when variations in virus and host population susceptibility occurred. However, this study is based on one metropolitan WWTP in southern France and may not be generalized to other contexts such as different catchments, rural areas, or regions with different testing policies or population dynamics. Future works could involve multi-site studies as previously prefigurated in France by the Obepine project (Cluzel et al., 2022).

## 5 Conclusion

The present study is one of the longest longitudinal studies of SARS-CoV-2 in wastewater published to date. Spanning 5.5 years, it captures different epidemiological trends of the SARS-CoV-2 pandemic, including the pandemic and post-pandemic phases. The study suggests that WS could be implemented permanently in order to continue surveillance regardless of the epidemiological phase of a viral infection: (i) when a virus or variant emerges and when individual diagnosis tools are lacking; (ii) during epidemic/pandemic periods to provide additional, cost-effective data for crisis management; (iii) during endemic/epidemic periods when public health policies do not prioritize individual diagnosis. This study and others suggest early warning of WS that could help prepare hospital and community response to epidemics. Continuing WS also provides opportunities for variant surveillance via virus genome sequencing or other innovative approaches (Li et al., 2026). Given the strong association between WS and non-specific syndromic indicators, such as the number of emergency procedures, it would be valuable to integrate these approaches to identify which circulating virus is driving increases in non-specific medical indicators. To this end, surveillance should be expanded to include viruses that significantly affect community health and are usually monitored via syndromic surveillance, such as respiratory (Ulloa-Medina et al., 2026) or gastrointestinal (Kuhn et al., 2023) pathogens.

## Data Availability

The raw data supporting the conclusions of this article will be made available by the authors, without undue reservation.

## Footnotes

1
https://www.legifrance.gouv.fr

2
https://covariants.org/

3
https://odisse.santepubliquefrance.fr
