# Supplementary material for: Long-term wastewater surveillance of SARS-CoV-2: a 5.5-year study reveals correlation with public health indicators and early warning potential during and after the pandemic
Source: Front Microbiol. 2026 Aug 18;17:1854041. doi: 10.3389/fmicb.2026.1854041 (PMC13529775; doi:10.3389/fmicb.2026.1854041)
Supplement: Supplementary file 1 [file Data_Sheet_1.docx]

SUPPLEMENTARY MATERIAL

**Long-term wastewater surveillance of SARS-CoV-2: a 5.5-year study reveals correlation with public-health indicators and early-warning potential during and after the pandemic**

Authors: Mahé Liabeuf^1,2^, Mylène Toubiana^1^, Charlotte Arnal^2^, Emmanuel Soyeux^2^, Vincent Foulongne^3,4^, Patricia Licznar-Fajardo^1,5^, Olivier Courot^6^, Patrick Monfort^1^, Estelle Jumas-Bilak^1,5^

**Affiliations :**

^1^ HSM, Univ Montpellier, CNRS, IRD, Montpellier, France

^2^ Veolia Scientific & Technological Expertise Department, Aubervilliers, France

^3^PCCEI, Univ Montpellier, CNRS, IRD, Montpellier, France

^4^Virology Laboratory, CHU de Montpellier, Montpellier, France

^5^ SPIR, CHU de Montpellier, Montpellier, France

^6^IAGE, Montpellier, France

**Supplementary figures 1 to 6**

**Supplementary Table 1**

**Supplementary Figure 1**: Distribution of PMMoV RNA quantities in Genome Units per liter (GU/L) after the two extraction protocols used in this study. Extraction protocol 1 was used on WW samples collected from the beginning of the study to the 13 February 2023, and extraction protocol 2 (EP2) on all the samples collected after this date. Comparison of distributions using a Mann-Whitney test indicates a significant difference on PMMoV quantities between the samples extracted with EP1 and 2. (****p-value ≤0.0001). The protocol 2 appears to have a greater sensibility with 12% increase of yield.

**Supplementary Figure 2**: Comparative dynamics of SARS-CoV-2 RNA loads in GU/day and concentrations of SARS-CoV-2 RNA (GU/L) normalized by concentrations of PMMoV/L (SARS-CoV-2/PMMoV ratio) from April 2021 to November 2025. The Spearman correlation coefficient (rs) was 0.91 (p≤0.001) indicating a very strong correlation.

**Supplementary Figure 3:** Dynamics of SARS-CoV-2 in GU/Day during the 65 months of the study period compared to the rate of positive individual tests in Hérault (red curve, before 1st June 2023) and the rate of positive individual tests in the regional hospital (orange curve, after 1st January 2023). Positivity rates are shown as a rolling weekly average.

**Supplementary Figure 4:** Comparative dynamics of the number of positives tests in Hérault (red curve, left y-axis) and in the University hospital of Montpellier (green curve, right y-axis) between January 2023 and July 2023. Number of positive tests are given as a rolling weekly average. The Spearman correlation coefficient (rs) between these two indicators was 0.799 (p<0.001) revealing a very strong correlation. The same test between the rate of positive tests in Hérault and in the University hospital of Montpellier gave similar results, with a very strong correlation (rs = 0.81, p<0.001) (data not shown).

**Supplementary Figure 5:** Comparative dynamics of SARS-CoV-2 RNA load in GU/day (blue curve), the number of positive tests (orange bars) and the incidence of positive tests (black curve) in Hérault in the pandemic period. The incidence of positive tests is given as a rolling weekly average. The blue background indicate the period corresponding to wave 4 to 7.

**Supplementary Figure 6:** Evolution of Spearman correlation coefficient (*r_s_*) according to day shift. Correlation coefficients are calculated on daily interpolated data between SARS-CoV-2 in GU/Day concentration in wastewater and the health indicators measured for the 8 waves numbered in the top of each graph. Lags were considered significant if the bootstrap p-values associated to the corresponding *r*_s_ were ≤0.05.

**Supplementary Table 1:** Characteristics and use of public-health indicators (n=15) tested in this study.

| **Public health indicators** | **Geographical scale** | **Data collection system** | **Availability period** | **Analysed**  **period** | **Analyses^2^** |
| --- | --- | --- | --- | --- | --- |
| Medical procedures | Hérault  (department) | Passive, continue surveillance  Syndromic  Non specific  *SurSaUD database* | 14 July 2020 to 13 November 2025 | 14 July 2020 to 13 November 2025 | Five years  Pandemic / post-pandemic  Before Omicron / Omicron  Before / After vaccination  8 individual waves |
| Emergency procedures |  |  |  |  |  |
| Post emergency hospitalization |  |  |  |  |  |
| Number of positive tests |  | Pandemic surveillance  Virology-based  Specific  *SI-DEP and SI-VIC database* | 14 July 2020 to 30 June 2023 | 14 July 2020 to  5 May 2023 | Pandemic period  Before Omicron / Omicron  Before / After vaccination  8 individual waves |
| Rate of positive tests |  |  |  |  |  |
| Positive tests incidence^1^ |  |  |  |  |  |
| R effective |  |  |  |  |  |
| Hospitalization incidence^1^ |  |  |  |  |  |
| Intensive care unit incidence^1^ |  |  |  |  |  |
| Intensive care unit occupancy rate |  |  |  |  |  |
| Death incidence^1^ |  |  |  |  |  |
| Positive tests rate | Montpellier  (city) | Internal hospital database  Virology-based  Specific | 1 January 2023 to 13 November 2025 | 5 May 2023 to  13 November 2025 | Post-pandemic period |
| Number of positive tests |  |  |  |  |  |
| Number of patients hospitalized with COVID-19 |  |  |  |  |  |
| Number of patients hospitalized for COVID-19 |  |  |  |  |  |

^1^for 100,000 inhabitants; ^2^see Table in main text for the dates of each analysis
